# Illness perception characteristics and influencing factors in adult patients with myasthenia gravis in China

**DOI:** 10.1002/brb3.2451

**Published:** 2021-12-12

**Authors:** Le Xu, Xia Wang, Yafeng Cui, Yanghua Tian, Nong Zhou, Juanjuan Zhang, Hiaxia Ji, Xiumei Cheng, Qin Zhang, Qingfeng Li, Panpan Hu, Long Zhang

**Affiliations:** ^1^ Department of Neurology The First Affiliated Hospital of Anhui Medical University Hefei Anhui Province China

**Keywords:** illness perception, influencing factors, myasthenia gravis, questionnaire, variance and multivariate linear regression

## Abstract

**Objective:**

We aimed to examine the illness perception characteristics of patients with myasthenia gravis and analyze the associated factors.

**Methods:**

A general information questionnaire, Illness Perception Questionnaire, and Simplified Coping Style Questionnaire were used to survey 90 patients with myasthenia gravis. One‐way analysis of variance and multivariate linear regression were used for statistical analysis.

**Results:**

The timeline (acute/chronic), consequence, and emotional representation scores of patients with myasthenia gravis were 18.31 ± 4.47, 18.58 ± 3.57, and 20.80 ± 4.56, respectively. Multivariate stepwise linear regression analysis showed that marital status, disease duration, educational level, disease type, and coping style are factors associated with illness perception in patients with myasthenia gravis.

**Conclusion:**

Patients who have myasthenia gravis have a negative illness perception. Medical professionals should provide targeted intervention measures based on the factors associated with illness perception.

## Abstract 2

There is a need to improve current illness perception scores among patients with myasthenia gravis. The results of this study showed that coping style, marital status, and educational level are important factors related to illness perception among patients with myasthenia gravis. Patients who have myasthenia gravis have a negative illness perception. Medical professionals should provide targeted intervention measures based on the factors associated with illness perception.

## 1 INTRODUCTION

Illness perception is the process by which a patient uses their existing knowledge and background to explain their current symptoms, including an individual's emotional characterization of the disease and psychological behavioral responses. The theoretical foundation of illness perception is the self‐regulatory model (SRM) proposed by Leventhal et al. (1998). A large number of Chinese and international studies on various diseases have found that illness perception directly or indirectly affects survival, disease prognosis, and emotional health (de Raaij et al., 2018; Presciutti et al., 2020; Solmaz et al., 2020). Timkova et al. (2021) and Wilski & Tasiemski (2016) showed that the timeline cyclical and treatment control in illness perception in patients with multiple sclerosis (MS) were significantly correlated with the self‐management of MS. Bogdan et al. (2020) pointed out that the emotional factor was a key risk factor for the recurrence of myasthenia gravis (MG). Chinese studies have shown that illness perception has direct and indirect effects on psychological resilience and can affect the quality of life of patients through emotional adjustment, coping style, and self‐efficacy (Wang et al., 2018; Liu et al., 2021; Wang, 2018).

MG is an autoimmune disease of the nervous system that is an autoantibody‐mediated acquired neuromuscular junction disorder. The global incidence rate is approximately 0.4−1.0 per 100,000, and the incidence rate in China is 0.68 per 100,000 (Chang, 2021). MG has diverse clinical presentations that are characterized by fatigue, a tendency for recurrence, and symptom fluctuations. MG has a long treatment cycle and often has severe psychological and physiological effects on patients (Li, 2016; Sanders et al., 2018). Chen et al. (2011) examined illness perception in patients with MG before and after plasmapheresis. The results showed that patients have a clearer cognition of the disease after plasmapheresis. However, these authors only considered the impact of treatment (i.e., plasmapheresis) on the illness perception of patients with MG. They did not assess additional factors, such as coping styles, that impact illness perception in patients with MG. In this study, we attempt to preliminarily examine illness perception in patients with MG, and the relationship between illness perception and coping styles to better guide clinical work and provide beneficial information for controlling disease outcomes.

## 2 PATIENTS AND METHODS

### 2.1 Study participants

A total of 102 patients with a definitive diagnosis of MG who were treated in the neurology department of the First Affiliated Hospital of Anhui Medical University from June 2018 to November 2020 were enrolled in this study. The inclusion criteria were: 1) age ≥ 18 years; 2) met the diagnostic criteria of the 2015 Chinese MG diagnosis and treatment guidelines; 3) stable disease; 4) possessed basic reading, writing, and comprehension skills and were able to complete the questionnaires independently; 5) provided written informed consent. Exclusion criteria were: 1) patients diagnosed with an acute progressive, advanced generalized weakness, or early muscular atrophy presentation based on the Osserman classification criteria; 2) patients with comorbid malignancy or severe physical disorder; 3) patients with mental or communication disorders; 4) patients who were unable to complete the questionnaires independently. A total of 102 questionnaires were distributed and 90 valid questionnaires were recovered. The valid response rate was 88.24%. Questionnaires were considered valid when 1) the patient completed all the questions, and 2) the answers of items from IP1 to IP38 on the illness perception questionnaire were not contradictory. The Clinical Research Ethics Committee of Anhui Medical University approved this study protocol, which was in accordance with the Declaration of Helsinki. Patients gave written informed consent.

### 2.2 Survey tools

The questionnaires used in this study included a self‐designed general information questionnaire, Chinese Illness Perception Questionnaire‐Revised (CIPQ‐R), and the Simplified Coping Style Questionnaire (SCSQ).

#### 2.2.1 General information questionnaire

This questionnaire was designed by the researchers to collect information on sex, age, marital status, educational level, family monthly income, disease type, and disease duration.

#### 2.2.2 CIPQ‐R (Ma et al., 2015)

This questionnaire contains three sections and 70 questions. Section 1 includes 14 questions on common symptoms and is used to assess illness identity. Section 2 contains 38 questions and is used to score seven dimensions (timeline [acute/chronic], consequence, personal control, treatment control, illness coherence, timeline cyclical, and emotional representation). The reliability of the eight dimensions were: 0.75, 0.84, 0.66, 0.80, 0.69, 0.76, 0.69, and 0.87.

#### 2.2.3 SCSQ (Xie, 1998)

We used the SCSQ as modified by Xie (1998). This questionnaire contains two dimensions, positive coping style (12 questions) and negative coping style (8 questions). The higher the positive coping style score, the more likely that the individual uses a positive coping style to solve problems. The higher the negative coping style score, the more likely that the individual uses a negative coping style to solve problems. The Cronbach's α of the positive coping style and negative coping style scales were 0.89 and 0.78, respectively, showing that the scales have good validity and reliability.

## 3 DATA COLLECTION

The researchers distributed the questionnaires to each individual patient within 24 h after admission. Before the questionnaires were distributed, the objectives and importance of the study were explained to participants and their family members. After consent was given by the patient and family, the questionnaires were completed independently by the patient within 20 min. The questionnaires were immediately verified and collected. Participants were asked to complete any missing items on the questionnaires.

### 3.1 Statistical analysis

EpiData3.1 was used for data entry and IBM SPSS 20.0 (IBM Corp., Armonk, NY, USA) was used for statistical analysis. Quantitative data are expressed as mean ± standard deviation. An independent samples *t* test was used to compare differences in illness perception between two groups of patients. Univariate correlation analysis was used to examine illness perception‐related factors among patients with MG, and multivariate stepwise linear regression was used to examine the risk factors of each illness perception dimension. A difference of *p* < .05 was considered statistically significant.

## 4 RESULTS

### 4.1 General status of included patients

The age of patients ranged from 21 to 75 years, and the mean age was 57.33 ± 12.22 years. Most participants were married and had a middle school education or above. Based on Osserman classification, patients with MG were classified as purely ocular, mild generalized, and moderately generalized. Details are shown in Table 1.

**TABLE 1:** General patient information

**Item**	***n***	**Percentage**
Gender (n)		
Male	54	60%
Female	36	40%
Age		
40 years and below	7	7.8%
41−50 years	15	16.7%
51−60 years	29	32.2%
> 61 years	39	33.3%
Marital status		
Married	82	91.1%
Others	8	8.9%
Educational level		
Primary school and below	14	15.6%
Middle school and above	76	84.4%
Family monthly income		
≤3000 RMB/month	24	26.7%
3001−5000 RMB/month	40	44.4%
>5000 RMB/month	26	28.9%
Disease duration		
0−1 year	30	33.3%
1−2 years	24	26.7%
2−3 years	12	13.3%
≥3 years	24	26.7%
Type		
Purely ocular	32	35.6%
Mild generalized	51	56.7%
Moderate generalized	7	7.7%

### 4.2 Current illness perception status of patients with myasthenia gravis (MG)

The timeline (acute/chronic), consequence, and emotional representation scores of patients with MG were 18.31 ± 4.47, 18.58 ± 3.57, and 20.80 ± 4.56, respectively, showing that patients had negative illness perception. Details are shown in Table 2.

**TABLE 2:** Illness perception questionnaire scores among patients with myasthenia gravis (*n* = 90)

**Item**	**Score range**	**`x ± s**
Illness identity	0−14	3.66 ± 2.22
Timeline (acute/chronic)	6−30	18.31 ± 4.47
Consequence	6−30	18.58 ± 3.57
Personal control	5−25	19.98 ± 3.37
Treatment control	5−25	18.09 ± 1.52
Illness coherence	5−25	14.31 ± 4.122
Timeline cyclical	4−20	12.49 ± 3.15
Emotional representation	6−30	20.80 ± 4.56

### 4.3 Comparative analysis of illness perception scores based on demographic characteristics

All enrolled patients were grouped according to general characteristics. The results of one‐way analysis of variance (ANOVA) showed that: illness identity score differences among patients of different education levels were statistically significant (*t* = 2.895, *p* < .05); timeline (acute/chronic) score differences were significant between patients according to marital status and coping style (*t* = 5.115, *p* < .01, *t* = 4.060, *p* < .05); differences in consequence factor score were statistically significant among patients with different disease types (*f* = 3.657, *p* < .05); differences in illness correlation scores were statistically significant among patients with different disease durations and coping styles (f = 2.802, *p* < .05, *t* = 11.823, *p* < .01); differences in timeline cyclical scores were significant according to sex (*t* = 5.910, *p* < .01); and differences in emotional representation scores were statistically significant among patients with different coping styles (*t* = 10.668, *p* < .01). There were no statistically significant differences in illness perception scores according to family monthly income (*p* > .05). See Table 3 for the results.

**TABLE 3:** Illness perception scores among patients with myasthenia gravis according to sociodemographic characteristics

**Item**	***n***	**Illness identity**	**Timeline (acute/chronic)**	**Consequence**	**Personal control**	**Treatment control**	**Illness coherence**	**Timeline cyclical**	**Emotional representation**
Gender (*n*)									
Male	54	3.57 ± 2.31	17.85 ± 4.67	18.11 ± 3.83	20.41 ± 3.59	18.33 ± 1.57	12.59 ± 4.12	11.85 ± 3.40	20.20 ± 5.01
Female	36	3.75 ± 2.02	19.00 ± 4.04	19.28 ± 3.00	19.33 ± 2.90	17.72 ± 1.37	13.56 ± 4.20	13.44 ± 2.42	21.69 ± 3.25
*t*‐Value		0.138	1.451	2.368	2.244	3.636	1.161	5.910**	2.487
Age (years)									
≤ 40	7	4.29 ± 2.75	19.14 ± 2.27	18.00 ± 4.20	19.00 ± 4.12	17.72 ± 0.76	16.86 ± 1.35	11.57 ± 3.74	18.29 ± 3.77
41−50	15	3.87 ± 1.64	19.27 ± 3.31	19.93 ± 3.52	18.53 ± 4.56	17.47 ± 1.06	15.00 ± 3.46	13.47 ± 2.85	20.67 ± 3.24
51−60	29	4.21 ± 1.90	19.17 ± 4.15	17.34 ± 3.11	19.97 ± 304	18.24 ± 1.64	12.83 ± 4.27	12.27 ± 3.24	20.83 ± 4.40
> 60	39	3.03 ± 2.78	17.15 ± 5.12	19.08 ± 3.59	20.72 ± 3.40	18.36 ± 1.58	11.62 ± 4.00	12.08 ± 3.04	21.28 ± 4.92
F‐statistic		1.993	1.591	2.311	1.805	2.095	5.294	0.987	0.908
Marital status									
Married	82	3.70 ± 2.24	17.99 ± 4.42	18.43 ± 3.46	20.07 ± 3.41	18.10 ± 1.42	12.78 ± 4.06	12.41 ± 3.18	20.68 ± 4.46
Others	8	3.13 ± 1.62	21.63 ± 3.29	20.13 ± 4.29	19.00 ± 3.78	18.00 ± 2.39	15.00 ± 4.87	13.25 ± 2.61	20.66 ± 4.14
*t*‐value		0.491	5.115**	1.680	0.744	0.030	2.104	0.517	0.642
Education level									
Primary school and below	14	3.47 ± 2.36	17.25 ± 4.98	19.00 ± 3.01	20.44 ± 3.11	18.00 ± 1.52	11.88 ± 3.71	13.06 ± 2.79	21.75 ± 4.51
Middle school and above	76	3.74 ± 2.11	18.90 ± 4.04	18.24 ± 3.80	19.722 ± 3.48	18.14 ± 1.52	13.59 ± 4.29	12.17 ± 3.28	20.28 ± 4.34
*t*‐Value		2.895*	0.317	1.472	0.931	0.170	3.596	1.683	2.318
Family monthly income									
≤ 3000 RMB/month	24	3.00 ± 1.95	19.10 ± 3.75	18.94 ± 3.53	19.65 ± 3.22	17.94 ± 1.29	13.90 ± 4.17	12.42 ± 2.84	21.94 ± 3.73
3001−5000 RMB/month	40	3.89 ± 2.19	18.67 ± 4.40	18.53 ± 3.72	19.83 ± 3.65	18.11 ± 1.49	12.72 ± 4.23	12.44 ± 3.55	20.81 ± 4.41
> 5000 RMB/month	26	4.13 ± 2.38	16.70 ± 5.11	18.17 ± 3.42	20.65 ± 3.08	18.26 ± 1.84	12.13 ± 3.96	12.65 ± 2.92	19.26 ± 5.01
F‐statistic		2.191	2.178	0.305	0.645	0.308	1.324	0.042	2.487
Disease duration									
0−1 year	30	4.47 ± 1.87	17.67 ± 4.28	17.60 ± 2.72	20.20 ± 3.70	18.53 ± 1.57	11.33 ± 3.87	11.93 ± 3.43	19.20 ± 4.52
1−2 years	24	3.46 ± 2.57	19.92 ± 3.30	19.83 ± 3.00	19.08 ± 3.86	18.00 ± 1.25	13.42 ± 3.90	11.67 ± 3.13	21.08 ± 4.10
2−3 years	12	3.33 ± 2.50	16.33 ± 4.89	18.50 ± 4.03	21.33 ± 2.93	17.50 ± 0.52	13.33 ± 3.60	13.17 ± 2.72	22.83 ± 4.76
≥ 3 years	24	2.96 ± 1.76	18.50 ± 5.07	18.58 ± 4.44	19.92 ± 2.34	17.92 ± 1.89	14.42 ± 3.54	13.67 ± 2.62	21.50 ± 4.05
F‐statistic		2.449	2.144	1.809	1.280	1.638	2.802*	2.286	2.500
Type									
Purely ocular	32	3.89 ± 2.19	18.67 ± 4.40	18.53 ± 3.72	19.83 ± 3.65	18.11 ± 1.49	12.72 ± 4.23	12.44 ± 3.55	20.81 ± 4.41
Mild generalized	51	3.46 ± 2.57	19.92 ± 3.30	19.83 ± 3.00	19.08 ± 3.86	18.00 ± 1.25	13.42 ± 3.90	11.67 ± 3.13	21.08 ± 4.10
Moderate generalized	7	3.13 ± 1.62	21.63 ± 3.29	20.13 ± 4.29	19.00 ± 3.78	18.00 ± 2.39	15.00 ± 4.87	13.25 ± 2.61	20.66 ± 4.14
F‐statistic		1.653	2.121	3.657*	1.290	1.277	1.115	2.076	1.892
Coping style									
Positive	58	3.59 ± 2.00	19.00 ± 3.80	18.45 ± 3.76	19.69 ± 3.24	18.14 ± 1.54	14.03 ± 3.85	12.38 ± 3.55	19.72 ± 4.38
Negative	32	3.75 ± 2.54	17.06 ± 5.25	18.81 ± 3.18	20.50 ± 3.56	18.00 ± 1.48	11.06 ± 4.06	12.66 ± 2.21	22.75 ± 3.86
*t*‐Value		0.114	4.060*	0.215	1.206	0.170	11.823**	0.198	10.668**

* *p* < .05;

** *p* < .01.

### 4.4 Multivariate linear stepwise regression analysis results of factors associated with illness perception

Illness perception factor scores were used as dependent variables for multivariate linear regression analysis based on the results of one‐way ANOVA. Sex (male = 1, female = 2), age (≤ 40 years = 1, 41−50 years = 2, 51−60 years = 3, ≥ 61 years = 4), and coping style (positive = 1, negative = 2) were used as independent variables. The results showed that illness identity was associated with educational level and patients with a middle school education or above had the lowest scores. Timeline (acute/chronic) was associated with marital status and coping style. The timeline (acute/chronic) scores of married patients were lower than those of patients with other marital statuses, and patients with positive coping styles had lower timeline (acute/chronic) scores than those with a negative coping style. Consequence was associated with disease type and patients with purely ocular MG had the lowest scores. The illness correlation score was correlated with disease duration and coping style: patients with a longer disease duration had higher illness correlation scores. Timeline cyclical was associated with sex and male patients had lower scores than female patients. Emotional representation score was associated with coping style and patients with a negative coping style had higher emotional representation scores. The results are shown in Table 4.

**TABLE 4:** Results of multivariate stepwise regression analysis of factors related to illness perception

**Illness perception factor**	**Associated factor**	**β**	**SE**	**Beta**	***t*‐Value**
Illness identity	Education level	−2.531	0.958	−0.273	−2.754*
Timeline (acute/chronic)	Marital status	3.637	1.608	0.234	2.262*
	Coping style	−1.938	0.962	−0.210	−2.015*
Consequence	Disease type	1.584	0.362	0.227	2.587*
Illness coherence	Age	−1.703	0.428	−0.391	−3.981*
	Coping style	−2.972	0.864	−0.344	−3.438*
	Number of years since diagnosis	0.958	0.355	0.276	2.699*
Emotional representation	Coping style	3.026	0.926	0.329	3.266*

Abbreviation: SE, standard error.

* *p* < .05;

** *p* < .01.

## 5 DISCUSSION

### 5.1 Illness perception in patients with myasthenia gravis (MG)

Increasingly more patients are diagnosed with MG due to advances in medical diagnostics and national policy requirements. The results of this study showed that patients with MG had a negative illness perception; therefore, attention should be paid to evaluating illness perception in patients. CIPQ‐R consequence and emotional representation scores in patients were 18.58 ± 3.57 and 20.80 ± 4.56, respectively, which were higher than those in a study by Gong et al. (2020). Gong et al. assessed illness perception in 98 patients with epilepsy, with consequence and emotional representation scores of 17.41 ± 5.22 and 21.73 ± 5.79, respectively. Our findings show that patients with MG have more negative feelings than patients with chronic neurological disorders, are unable to integrate into society and assume corresponding social roles, and believe that their illness has a huge impact on their lifestyle and health, which increases the emotional effect of MG on the patient. Our findings are consistent with findings from Vitturi et al. (2021), who surveyed the psychosocial functioning of patients diagnosed with MG. Vitturi et al. found that the majority of the patients surveyed experienced a decrease in occupational functioning, had limited financial support outside of previous employment, and had the perception of having a limited social support system.

Personal control and treatment control scores in our patients were 19.98 ± 3.37 and 18.09 ± 1.52, respectively, which were lower than those in studies containing patients with chronic health conditions (Ji et al., 2014; Cao et al., 2007)). Ji et al. investigated 60 patients with ischemic stroke and obtained personal control and treatment control scores of 21.45 ± 3.08 and 18.88 ± 2.53, respectively. Cao et al. conducted a study among 87 patients with chronic hepatitis B virus infection. Personal control and treatment control scores were 20.64 ± 3.68 and 18.26 ± 3.01, respectively. These findings illustrate that individuals have limited means of obtaining disease‐related knowledge, have lower satisfaction towards treatment results, lack clear disease awareness, and have poor self‐confidence and disease control confidence. Compared with the results of disease identity, timeline (acute/chronic), consequence, timeline cyclical, and emotional representation in patients with type 2 diabetes in a study by Li et al. (2020), our patients with MG felt that the disease duration was long, that they will not substantially improve with time, held pessimistic views regarding their future, and had greater negative illness perception, and higher psychological stress. Negative emotions have an enormous impact on disease progression, prognosis, and quality of life (de Rooij et al., 2018; Minshall et al., 2020). Therefore, there is an urgent need to improve the illness perception of patients with MG.

### 5.2 Factors affecting illness perception of patients with MG

Illness perception scores represent different beliefs that patients have towards disease. The results of this study showed that educational level is an important factor in illness identity among patients with MG. Illness identity mainly refers to an individual's perception and understanding of disease symptoms and of stigma (Jiang et al., 2010). Patients with lower educational levels tend to be more affected by stigma regarding the disease and have poorer understanding of their disease symptoms. The reason for this is that patients who are less educated have fewer means of obtaining disease‐related information as well as limited acceptance and understanding of such information, resulting in poorer awareness of disease symptoms. Similar conclusions were reached in a Chinese study on illness perception in patients with chronic prostatitis and ischemic stroke (Jiang et al., 2010). Thus, there is a need to improve disease‐related knowledge for both the patient and their family and to develop different types of health education materials for patients according to educational level. The education protocol should be progressively revised based on differences in patients’ understanding of disease to improve understanding and knowledge among patients and family members.

The results of this study showed that coping style and marital status are important factors associated with timeline (acute/chronic) in patients with MG. Timeline (acute/chronic) refers to an individual's belief regarding the relatively long disease duration (Jiang et al., 2010). In China, there are no studies to date showing a correlation between marital status and timeline (acute/chronic). The results of this study showed that married patients firmly believe that MG is a transient process. Marriage is an important component of social support, and poor social support tends to lead to incorrect understanding of the disease duration among patients (Hoseini et al., 2016; Nedjat‐Haiem et al., 2020; Recto & Champion, 2020). A possible reason for this may be that married patients have stronger social and family support than unmarried individuals. Negative coping style leads patients to believe that MG is a long‐term disease and a long disease duration exacerbates negative emotions in patients. High medical costs place an immense burden on patients’ families. At the same time, impaired mobility causes psychological and financial stress for patients, causing them to feel guilt, self‐blame, and other negative emotions. This leads to anxiety and depression and a pessimistic view in the patient. Hence, coping style and negative emotions mutually affect each other. Therefore, clinical staff should pay greater attention to this population to increase social support in patients, conduct further investigations to analyze the causes of negative coping responses, carry out target intervention together with the patient's family, and correct erroneous disease knowledge.

The higher the illness correlation factor, the better the patient's understanding of the disease. The results of this study showed that disease duration and coping styles are factors associated with illness coherence. Illness correlation reflects an individual's understanding of the disease itself (Jiang et al., 2010). The longer the disease duration, the greater the patient's disease understanding. Patients with MG tend to experience relapses and undergo long‐term medical treatment, so they have a good understanding of the disease. Patients with negative coping styles have a higher illness correlation score. As MG has a long disease duration, patients with better understanding of the disease tend to understand that this disease is a chronic neuroimmune disorder with low probability of cure, and they will have to live with the disease for the rest of their life. This worsens negative emotions in the patient, resulting in a negative coping style. Additionally, as medical technology advances, disease understanding deepens. The 2020 Chinese MG diagnosis and treatment guidelines point out that the best treatment status is to achieve minimal disease status or better and grade ≤ 1 treatment‐related side effects (Chang, 2021), for patients to be able to live more harmoniously with the disease for a long period of time and achieve a body–disease equilibrium. Most patients do not have good routes through which to obtain the latest medical knowledge and public information dissemination is not ideal. Therefore, national health authorities can develop information dissemination platforms such as public WeChat accounts, television ads, and video clips, as well as relevant guidelines for medical professionals, to increase public awareness about MG.

In this study, we found that coping style is the main factor associated with emotional representation. Emotional representation mainly reflects an individual's awareness of abnormal negative emotions after developing a disease (Jiang et al., 2010). Patients with a negative coping style have higher emotional representation scores, indicating that they have more negative emotions. Negative coping styles increase negative emotions in patients, such as depression and anxiety, which is consistent with previous studies (Ji et al., 2016). A study on illness perception in patients with epilepsy found that negative emotions affect patients’ social role and quality of life. This suggests that medical professionals must apply different measures based on the patient's condition to decrease the emotional representation score.

## 6 CONCLUSIONS

There is a need to improve current illness perception scores among patients with MG. The results of this study showed that coping style, marital status, and educational level are important factors related to illness perception among patients with MG. Finally, coping style and social support were identified as two major factors. We did not examine the relationship between illness perception and social support in this study and did not further analyze the reasons for coping styles. In future studies, the sample size can be increased and an analysis of factors related to illness perception among patients with MG should be carried out to provide clearer information for medical staff regarding targeted and precise measures that will enable patients to cope with the disease in their best state, thereby decreasing disease recurrence, improving quality of life, and increasing illness perception among these patients. Furthermore, the relationship between illness perception and treatment is also worth studying in the future.

## CONFLICT OF INTEREST

None of the authors has any conflicts of interest to disclose.

### PEER REVIEW

The peer review history for this article is available at https://publons.com/publon/10.1002/brb3.2451

## Data Availability

The data that support the findings of this study are available from the corresponding authors upon request.
